# Carotid Body and Metabolic Syndrome: Mechanisms and Potential Therapeutic Targets

**DOI:** 10.3390/ijms21145117

**Published:** 2020-07-20

**Authors:** Lenise J. Kim, Vsevolod Y. Polotsky

**Affiliations:** Division of Pulmonary and Critical Care Medicine, Department of Medicine, School of Medicine, Johns Hopkins University, Baltimore, MD 21224, USA; vpolots1@jhmi.edu

**Keywords:** carotid body, metabolic syndrome, hypertension, glucose metabolism, sleep-disordered breathing, obesity, leptin, pharmacotherapy

## Abstract

The carotid body (CB) is responsible for the peripheral chemoreflex by sensing blood gases and pH. The CB also appears to act as a peripheral sensor of metabolites and hormones, regulating the metabolism. CB malfunction induces aberrant chemosensory responses that culminate in the tonic overactivation of the sympathetic nervous system. The sympatho-excitation evoked by CB may contribute to the pathogenesis of metabolic syndrome, inducing systemic hypertension, insulin resistance and sleep-disordered breathing. Several molecular pathways are involved in the modulation of CB activity, and their pharmacological manipulation may lead to overall benefits for cardiometabolic diseases. In this review, we will discuss the role of the CB in the regulation of metabolism and in the pathogenesis of the metabolic dysfunction induced by CB overactivity. We will also explore the potential pharmacological targets in the CB for the treatment of metabolic syndrome.

## 1. Introduction

Metabolic syndrome affects approximately one-fifth of the US adult population [1,2,3]. It was originally described as a set of metabolic abnormalities that co-exist in the same individual with a high frequency [4,5,6]. There are different definitions of this condition, but nearly all of them include glucose intolerance, insulin resistance, dyslipidemia and hypertension [4]. This cluster is associated with a high-risk of cardiovascular disease and all-cause mortality [7]. The pathophysiology of metabolic syndrome is still not entirely clear, but an overactivation of the sympathetic nervous system (SNS) appears to play a critical role, since it is observed in most of the metabolic disturbances that compose metabolic syndrome [8]. Investigators proposed that there is a single peripheral sensor for multiple metabolic parameters [9], dysfunction of which may lead to metabolic dysregulation and the impaired blood–brain barrier transport of metabolites in obese subjects [10,11,12,13,14,15,16,17]. Multiple investigators attributed this peripheral sensing function to the carotid body (CB).

The CB is a small organ located bilaterally at the bifurcation of the internal and the external common carotid arteries. The CB is morphologically organized in glomeruli, which are mainly composed of neuron-like glomus (type I) cells and glia-like type II cells [18]. Glomus cells (type I) are peripheral chemoreceptors with properties similar to sensory neurons and an ability to sense changes in arterial blood gases and pH. Type II cells were first described as supportive elements of CB glomeruli, being also called “sustentacular cells” [18]. More recently, it has been shown that type II cells are also involved in the CB plasticity and chemosensory response [18,19,20]. The CBs mainly govern the peripheral chemoreflex response and play a critical role in the control of breathing. The ventilatory response to hypoxia (HVR) is initiated by the CB, whereby low arterial O_2_ causes the depolarization of type I cells, and neurotransmitter release induced by a rise in intracellular Ca^+2^ and the closure of K^+^ channels [21]. Afferent chemosensory inputs from glomus cells reach the brainstem respiratory network via the carotid sinus nerve (CSN), a branch of the glossopharyngeal nerve, which projects into the nucleus of the solitary tract (NTS) and respiratory motoneurons, inducing hyperventilation [22,23,24,25,26].

Besides its well-known role in the peripheral chemoreflex and respiratory control, CB has been linked to metabolic regulation. Overactivation of CB has been demonstrated in cardiometabolic diseases, including hypertension, obesity and diabetes [27,28,29]. Exaggerated peripheral chemosensitivity in CB exacerbates SNS activity and promotes severe autonomic imbalance, which contributes to a poor prognosis in patients with chronic heart failure (HF) [30]. CB glomus cells also express a large number of genes of the G protein-coupled receptor signaling pathways [31], suggesting the sensing of the numerous metabolites in glomus cells and consequent paracrine modulation of CB [18]. Emerging evidence suggests that CB is not merely a hypoxia and pH sensor, but rather a multimodal sensor, responsive to different metabolic and hormonal stimuli, such as glucose, insulin and leptin [32]. In this review, we will explore the role of CB chemosensory activity in the regulation of metabolic homeostasis. We will review the available evidence on the involvement of CB malfunction in the pathogenesis of different components of metabolic syndrome mediated by tonic overactivation of SNS. Finally, we will provide insights into new potential pharmacological targets in the CB for the treatment of cardiometabolic dysfunction.

## 2. Hypoxia and Metabolic Dysfunction: What Is the Role of CB?

Hypoxia is the main stimulus for CB chemosensory activity. In sustained exposure to hypoxia observed at high altitude, CB plays a fundamental role in the development of cellular and neurochemical rearrangements that culminate in hypoxic ventilatory acclimatization (HVA) [33]. On the other hand, such acclimatization does not occur during intermittent hypoxia (IH), which leads to sympatho-excitation and cardiometabolic morbidity [24,34].

IH is the main factor in the pathology of obstructive sleep apnea (OSA) [35]. OSA is a sleep-disordered breathing, characterized by cyclical interruptions of the airflow during sleep due to partial or complete obstructions of the upper airway [35]. OSA is associated with a high risk of cardiometabolic diseases, including arterial hypertension and type 2 diabetes [36,37], suggesting the negative effects of chronic IH on metabolism. In fact, our research group has been provided plenty of evidence on the causal role of IH in metabolic disturbances in rodents [38,39,40,41,42,43,44,45,46,47,48,49]. Studies from our laboratory have shown a causal role of IH in the dysregulation of the glucose metabolism. Lean C57BL/6J mice exposed to acute IH had reduced whole-body insulin sensitivity and glucose utilization in their oxidative muscle fibers [38]. Similar findings were observed in diet-induced obese mice (DIO) and in leptin-deficient obese mice, in which chronic IH enhanced glucose intolerance and increased fasting serum insulin levels in a time-dependent manner [39,40]. IH-induced glucose intolerance was also abolished by adrenal medullectomy [41,42]. We have shown that IH impairs lipids metabolism. IH increased triglyceride concentrations and reduced the clearance of triglyceride-rich lipoproteins [43,44,45,46,47], which appeared to be related to an overexpression of stearoyl coenzyme A desaturase 1 (SCD-1) and atherosclerosis development [48]. Finally, our group has also shown that IH elevated mean arterial blood pressure, and exacerbated dyslipidemia and atherosclerosis in Apolipoprotein E-deficient (*ApoE^−^*^/−^) mice [49]. Taken together, this evidence suggests the involvement of OSA-induced IH in the pathology of metabolic syndrome, partially explaining the high prevalence of metabolic dysfunctions in apneic patients.

Available evidence from studies using IH protocols has indicated that IH leads to metabolic disturbances, but also has raised the following secondary questions: (1) could IH affect CB activity?; (2) is CB involved in the IH-mediated metabolic regulation?; if so, (3) how does CB contribute to the IH-induced metabolic disorders? Dr. Nanduri Prabhakar’s laboratory comprehensively examined the effect of IH on the CB activity. Chronic exposures to IH (5% O_2_) evoked a 48% increase in HVR in rats [50]. The investigators have also shown the contribution of CB to persistent, IH-induced, sustained increases in ventilatory neural activity, called long-term facilitation (LTF). Chronic IH induced LTF in the CB [51], possibly via mechanisms involving reactive oxygen species (ROS), mitochondrial-to-membrane signaling [52], alterations in the balance of gasotransmitters [53] and hypoxia-inducible factors 1 and 2 alpha (HIF-1α and -2α) pathways [54,55]. These data suggest that IH can produce functional plasticity in CB, and enhance peripheral chemosensitivity by multiple possible mechanisms. Given that CB stimulation results in SNS activation, and SNS plays a role in glucose tolerance and insulin resistance, our group has tested whether IH-induced glucose metabolism impairment could be modulated by CB. Shin and collaborators [56] had shown that IH–induced glucose intolerance in mice was abolished by the bilateral CSN denervation, supporting the role of CB in metabolic responses to hypoxemia. Of note is the fact that other groups have also reported the beneficial effects of CB resection on IH-induced hypertension [57,58]. Hence, CB contributes to the cardiometabolic dysfunction induced by IH, and the ablation of CB activity may normalize the metabolism in response to low arterial O_2_ levels. The mechanisms by which CB controls the metabolism at hypoxic conditions are still elusive, but overactivation of the SNS may play a crucial role. 

## 3. CB and Sympathetic Activity: A Common Way to Metabolic Dysfunction

**Figure 1:**
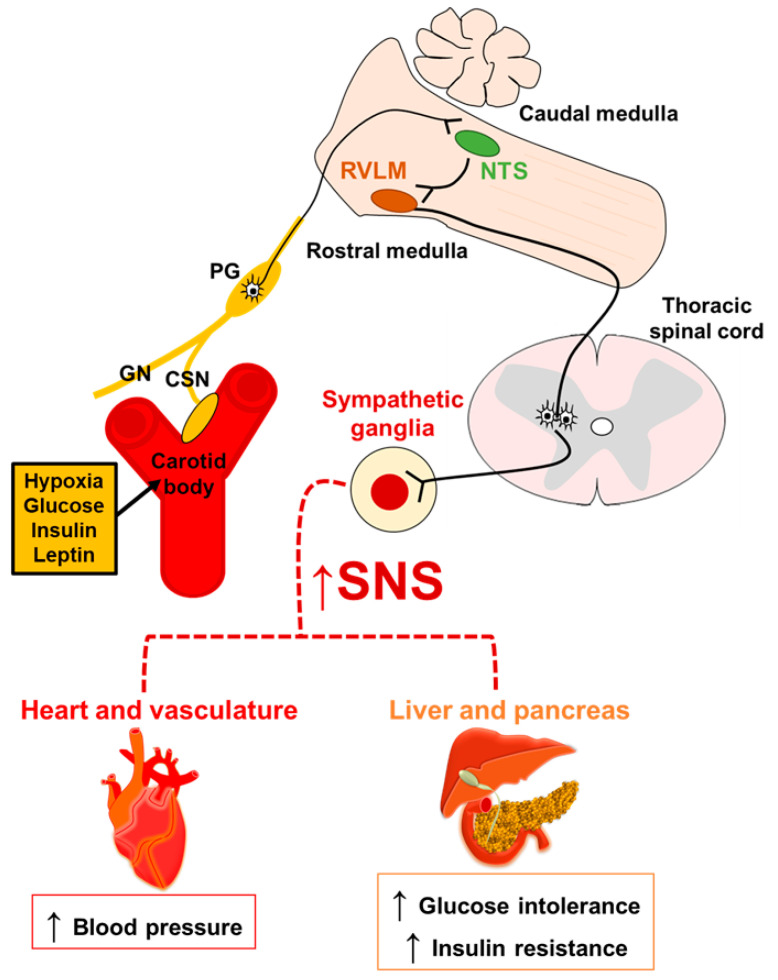
Mechanisms by which carotid body activation increases blood pressure and leads to metabolic abnormalities. Hypoxia, glucose, insulin and leptin activate the carotid body, which provides the afferent chemosensory input to the nucleus of the solitary tract (NTS) via the glossopharyngeal nerve (GN) subsequently activating neurons in the rostral ventrolateral medulla (RVLM) and sympathetic ganglia, eliciting cardiovascular and metabolic disturbances. CSN: carotid sinus nerve; PG: petrosal ganglia; SNS: sympathetic nervous system.

An overactive SNS is a common feature of different components of metabolic syndrome [8,59]. Since hypoxia is associated with cardiometabolic diseases and modulates CB sensitization [51,52], it may act in the CB to activate SNS and lead to metabolic disturbances (Figure 1). 

Hypoxia is implicated in the overactivation of SNS. Rats exposed to IH show increased sympathetic nerve activity in different nerve preparations [60,61,62,63]. CB chemosensory projects into NTS and thereafter to the respiratory medullary centers, resulting in increased sympathetic output. This involvement of sympathetic activity in the peripheral chemoreflex has been initially shown in anesthetized dogs, in which the carotid chemoreceptor regulated renal hemodynamics via renal sympathetic nerves [64]. The long-term up-regulation of CB induced by chronic IH exacerbates sympathetic activity [34,65]. Indeed, the sympathetic activity stimulated by the hypoxic chemoreflex is augmented in patients with OSA, as indicated by the increased HVR associated with elevations in blood pressure [66,67]. OSA increases baseline muscle sympathetic nerve activity (MSNA), and leads to a greater increase in MSNA in response to acute hypoxia, compared to healthy individuals [68] or metabolic syndrome patients without OSA [69]. Moreover, OSA treatment with continuous positive airway pressure (CPAP) decreased the ventilatory responses to hypoxia and partially normalized the MSNA [68,70]. In summary, these findings support the conclusion that CB chemosensitivity mediates the sympatho-excitation in OSA patients. 

As extensively reviewed by other authors (for more details, see [34,65]), the overactivation of SNS by IH-induced CB stimulation occurs at two different levels: (1) the chemosensory afferent input to NTS, and (2) the sympathetic output (Figure 1). Chronic IH increases neuronal activation in NTS areas related to chemosensory inputs [60]. IH induces excitatory postsynaptic activity in the NTS, regulating the expression of glutamatergic receptors [71,72,73]. Chemosensory input is processed in the NTS and then projected to the respiratory medullary centers, including the rostral ventrolateral medulla (RVLM) [74]. In the RVLM, IH increases the excitatory synaptic input in a subpopulation of presympathetic neurons [75], which suggests a possible neural signaling pathway for the IH-induced SNS overactivation. IH may also act on SNS peripherally by regulating the release of adrenal medulla catecholamines, but the results are still inconsistent in the literature [76,77]. Taken together, this evidence suggests that exaggerated CB responses to arterial O_2_ levels may induce a common pathology for different components of metabolic dysfunction: an overactive SNS. Thus, hypertension, impairments in glucose metabolism, insulin resistance and obesity could be the results of chronic sympatho-excitatory malfunctions of CB chemosensory activity.

## 4. Metabolic Syndrome and CB Chemosensory Response

The sympathetic activation induced by the CB chemoreflex may play an important role in the pathogenesis of metabolic syndrome. It is conceivable that CB is involved in the normal control of the metabolism, regulating glucose levels, insulin tolerance and blood pressure. A key point in this discussion is whether CB directly senses metabolic markers, or modulates the metabolism by activating secondary pathways. Regardless, CB may act as a sentinel organ, detecting fluctuations in homeostasis and evoking counterregulatory responses in the presence of metabolic distress. Here, we will discuss the current evidence on the homeostatic function of CB, emphasizing the main components of metabolic syndrome.

### 4.1. Hypertension and CB Chemoreflex

The stimulation of the CB by hypoxia triggers a series of cardiorespiratory responses, including hyperventilation and increased sympathetic activity, to restore the normal balance in arterial blood gases [22]. Cardiovascular reflexes induced by CB stimulation depend on the magnitude of the respiratory response [78]. Hyperventilation mediated via the CB chemoreflex comprises an afferent signal that activates the pulmonary stretch receptors (Hering–Breuer reflex). The augmented breaths are also associated with a phase of cardiac vagal withdrawal and increased sympathetic tone, leading to transient tachycardia and vasoconstriction [78,79,80]. It appears that the CB plays an important role in cardiovascular homeostasis, and exaggerated stimulus or CB malfunction in response to hypoxemia are involved in the development of systemic hypertension.

Systemic hypertension induces alterations in the CB structure. In humans, hypertension and HF are significantly associated with the CB enlargement detected by ultrasonography [81]. An enlarged CB, vascular expansion and glomus cells hypertrophy are also observed in spontaneously hypertensive (SH) rats compared to normotensive controls [82,83,84]. The available evidence suggests that the peripheral chemoreflex is augmented in hypertensive individuals. These findings were observed in both patients with hypertension and animal models of spontaneous hypertension. Somers and collaborators [85] have shown that borderline hypertensive subjects have enhanced chemoreflexes and a two-fold increase in sympathetic activity during hypoxia, compared to normotensive individuals. In animal models, differences in the cardiorespiratory response to the peripheral chemoreflex are strain-related. Increased ventilatory and sympathetic responses to hypoxia are observed in SH rats compared to normotensive strains [53,86,87,88]. The hypersensitivity of the peripheral chemoreflex can also be observed prior to the onset of hypertension. Augmented vascular resistance and respiratory-related bursts of sympathetic activity are already present in neonate SH rats compared to normotensive controls [89]. In the isolated CB glomus cells from young SH rats (before the hypertension onset), increased depolarization induced by low pH and elevated sympathetic nerve activity in response to chemoreceptors stimulation were observed, compared to control normotensive glomus cells [87]. The role of the CB in the regulation of blood pressure is also supported by evidence from studies using different techniques of CB activity ablation [78]. Hyperoxia exposures (100% O_2_) decreases blood pressure and sympathetic activity, measured by MSNA recordings, in patients with hypertension [90]. CB resection also improves blood pressure and causes a sustained sympatho-inhibition in both humans and animal models of hypertension [58,91,92]. 

The mechanism that regulates the hypersensitivity of the CB in hypertension is still unclear. In vitro, the differences in the chemoreceptors’ responsiveness to low pH, and a greater sympatho-excitation in SH rats, were associated with an overexpression of the two-pore domain acid sensing K^+^ channel (TASK1) in type I cells [87]. These results may suggest that the TASK channels family could be involved in the molecular pathway linking the CB chemoreflex response and hypertension. However, several studies have shown that the genetic ablation of TASK channels, specifically the subtypes TASK1 and TASK3, in knockout mice did not significantly change the ventilation in response to hypoxia and normoxic hypercapnia [93,94]. A normal hypoxic and hypercapnic responsiveness were also observed in TASK1/3-null glomus cells [94], and the absence of TASK channels did not impact the central respiratory chemosensitivity of the retrotrapezoid nucleus (RTN) [95]. Thus, the involvement of TASK channels in CB chemoreflex-induced hypertension appears to be minimal, and other molecular pathways could better explain this mechanism.

Our group proposed that CB induces hypertension by the stimulation of leptin signaling in the glomus cells, and the downstream activation of transient receptor potential melastatin 7 (TRPM7) channels. We have identified that the long-isoforms of leptin receptors (LepR^b^) are colocalized with TRPM7 channels in glomus cells, and that the overexpression of leptin receptors in LepR^b^-deficient *db/db* mice increased TRPM7 gene expression [96]. Moreover, systemic leptin infusions increased CSN activity *in vivo* in response to hypoxia and induced a nonselective cation current in glomus cells, which were both abolished by TRPM7 blockers [96,97,98]. Recently, Shin and collaborators [96] have shown that leptin induces hypertension by elevating the blood pressure in lean mice, when administered subcutaneously, and in LepR^b^-deficient *db/db* mice via the expression of leptin receptors in CB. Hypertension in both models was abolished by bilateral CSN resection and the blockade of TRPM7 signaling, either by the administration of a nonspecific TRPM7 blocker, FTY720, or by a viral vector containing short hairpin RNA. Taken together, our work supported the inference of the role of the CB in the regulation of blood pressure and in the pathogenesis of hypertension. Our findings have indicated that this modulation is mediated by the leptin signaling pathway in the CB, promoting the elevation of CB chemosensory activity and the consequent increase in blood pressure through the activation of TRPM7 channels. The potential involvement of leptin in enhancing the sympatho-excitation of the CB chemoreflex, and how leptin modulates the TRPM7 channels at a molecular level, are the focus of our current investigations. 

Interindividual variations in the cardiorespiratory response to the peripheral chemoreflex are observed among subjects, and may highlight the potential mechanisms for different CBs sensing O_2_ and its consequences on blood pressure. One possible mechanism was attributed to carbon monoxide (CO)-sensitive hydrogen sulfide (H_2_S) signaling in CB [53]. In SH rats, but not in normotensive controls, CBs were hypersensitive to hypoxia, and had lower levels of CO and increased H_2_S generation. The inhibition of cystathionine-γ-lyase (CSE), an H_2_S-catalyzing enzyme, improved the CB’s response to hypoxia and reduced the blood pressure in SH rats. Other molecular pathways have also been shown to be related to differences in cardiorespiratory responses to the CB chemoreflex, being potential pharmacological targets for hypertension. Disruptions in the normal homeostatic balance between HIF-1α-dependent prooxidants and HIF-2α-dependent antioxidants in the CB induce oxidative stress and exacerbate SNS activity, one of the major determinants of hypertension [99]. The activation of angiotensin II signaling and decreased nitric oxide synthase were also associated with an augmented CB chemoreflex in a rabbit model of HF [100,101]. 

### 4.2. Glucose Metabolism and CB Chemoreflex

The available evidence, especially from studies with animal models of IH (see item 2. Hypoxia and metabolic dysfunctions: what is the role of CB?), has pointed to the possible effect of the peripheral O_2_-sensing response on the regulation of blood glucose levels, and a possible role of CB in the pathophysiology of diabetes. Morphological and structural changes in the CB can also be observed in animal models of insulin resistance and diabetes [102,103], showing that CB is enlarged in these conditions. There is an ongoing discussion of the ability of the CB to directly sense blood glucose levels, as well as a potential role of CB overactivation in the pathogenesis of insulin resistance and diabetes. 

#### 4.2.1. Does CB Directly Sense Glucose Levels?

The pioneering study of Alvarez-Buylla and Alvarez-Buylla [104] has started the discussion about the CB sensing glucose levels, and its role in the control of glucose metabolism. The infusion of glucose into the isolated carotid sinus region of anesthetized cats depressed CB electrical activity by 20%, whereas the lack of glucose in the CB perfusate enhanced the chemosensory discharges, suggesting that changes in glucose levels may affect the peripheral chemoreceptor activity. Pardal and López-Barneo [105] have shown that low glucose concentrations increase the secretion of cathecolamines from CB glomus cells in an extracellular Ca^2+^-dependent manner, and by the inhibition of voltage-gated K^+^ channels. Other studies using CB ablation or hyperoxia have also supported the role of CB in physiological responses to hypoglycemia. In dogs, the bilateral resection of CB impaired the counterregulatory responses to hypoglycemia, reducing levels of cortisol and glucagon [106]. In humans, low glucose levels cause a robust increase in the concentration of the counterregulatory hormones glucagon, adrenaline, noradrenaline and cortisol, and enhance both isocapnic and hypoxic ventilation [107]. Systemic hyperoxia significantly impaired the counterregulatory hormone responses during hypoglycemic clamps in healthy patients [108]. Hypoglycemic clamps in healthy individuals also reduced HVR and spontaneous cardiac baroreflex sensitivity [109]. However, hyperoxia did not reverse the reductions in HVR or the baroreflex sensitivity induced by hypoglycemia, indicating that these changes during hypoglycemia are not exclusively attributed to the CB’s chemoreceptors. Taken together, these findings indicate the involvement of CB in the counterregulatory responses to hypoglycemia, which is especially important in cases of diabetic patients under insulin treatment, and suggests that CB malfunctions could be associated with the development of type 2 diabetes.

Although the current evidence has shown that CB regulates the glucose metabolism in response to hypoglycemia, there is still no consensus about CB’s ability to directly sense glucose. CB glomus cells responded to acute physiological decreases in glucose levels *in vitro*, shown by the increased secretion of catecholamines, as reported by Pardal and López-Barneo [105], and the augmented afferent neural discharge in the coculture of glomus cells and petrosal neurons [110]. However, evidence of the glucose sensitivity of the whole CB has not been consistent, and a lack of chemoreceptor discharge in response to low levels of glucose has been reported [111,112,113]. In addition to methodological differences that may have impacted the metabolic viability of CB preparations, a study by Holmes and collaborators [114] has proposed that the CB’s responses to hypoglycemia are related to glycogen metabolism. In an *in vitro* isolated rat CB preparation, the investigators showed that glycogen granules and enzymes related to glycogen conversion were expressed in the cytoplasm of type I cells, and the neighboring type II cells. The acute exposure to low glucose concentrations did not change the sensory neuronal discharge of CB, but the glycogen depletion decreased the time of physiological response to glucose deprivation by 65%. These findings may suggest that glycogen maintains CB chemoafferent activity during episodes of metabolic stress induced by hypoglycemia, by providing the energetic substrate from glycolysis. It may also indicate that acute glucose deprivation augments the CB’s responses to hypoxia, but a chronically low-glucose environment in CB may lead to an impaired ability to sense hypoxia [115]. More important evidence has been provided by Thompson and colleagues [116]. In Wistar rats, insulin-induced hypoglycemia augmented minute ventilation and CO_2_ sensitivity, but these effects were abolished by the blocking of the sympathetic pathways. These results indicate that the CB may not sense glucose levels directly.

Regardless of the mechanism, there is conclusive evidence that CB plays an important role in the counterregulatory response to hypoglycemia. Zhang and collaborators [110] have shown the synergistic effect of hypoxia and hypoglycemia on CB’s chemoafferent input, suggesting that low glucose stimulates CB via mechanisms similar to the hypoxic chemoreflex. Low glucose and O_2_ sensing in CB share similar signaling pathways, mediated by both the inhibition of voltage-gated K^+^ channels and the release of excitatory neurotransmitters in an extracellular Ca^2+^-dependent manner, leading to postsynaptic autonomic firing in the medullary centers [105,117,118]. However, in the case of low glucose levels, the initial depolarization of the CB also depends on the activation of Na^+^-permeable channels [117]. 

For many years, different hypotheses of how CB senses hypoxemia and pH have been proposed, but the exact mechanisms are still speculative. The involvement of gasotransmitters [53,115], olfactory receptor Olfr78 activated by lactate [119,120], and mitochondrial-to-membrane signaling [121,122] are some of them. Recently, the model of mitochondrial-to-membrane signaling was extensively reviewed by Ortega-Sáenz and López-Barneo (see [18]). Briefly, this model postulates the involvement of mitochondrial metabolism and the mitochondrial electron transport chain (ETC) system in the process of the depolarization of the CB glomus cells in a Ca^+2^- and K^+^-dependent manner [18,121]. The CB’s counterregulatory responses to low glucose could be related to this mitochondrial regulation. However, in a single CB cell preparation from Ndufs2-null mice, a knockout mouse for mitochondrial complex I (MCI) genes, the glomus cells lost their sensitivity to changes in PO_2_, but maintained a normal function in response to a glucose deprivation challenge [123]. In this sense, the control of glucose metabolism by the CB may occur via the activation of alternative pathways, related to the modulation of the CB’s sensing of O_2_ and the afferent chemoreflex response. Our group suggested that leptin signaling in the CB may also participate in the regulation of glucose metabolism.

Our group has previously demonstrated that the denervation of the CB abolished the glucose intolerance induced by IH [56]. As discussed above (item 4.1. Hypertension and CB chemoreflex), we have also shown that leptin stimulates CB glomus cells and augments CSN activity through TRPM7 channels [96,97,98], elevating blood pressure. In the *in vivo* electrophysiology of lean mice, Shirahata and collaborators [97] have confirmed the evidence that low glucose does not modify CSN activity. However, systemic leptin infusion was able to increase the CSN’s response to hypoxia, which was reversed by the administration of a TRP channel blocker. Supporting this potential role of TRP channels in the CB’s control of glucose metabolism, a previous study have demonstrated that low glucose levels stimulate CB transmitters release, possibly through the activation of a subtype C of TRP channels [117]. Taken together, this evidence has indicated the role of leptin signaling in the modulation of the CB chemosensory response, and support our hypothesis that CB indirectly senses low glucose levels by the activation of TRP channels (TRPM7?), mediated by leptin.

#### 4.2.2. Insulin: A Better Marker of CB-Induced Metabolic Dysfunctions?

It is well established that hypoglycemia, induced by the hyperinsulinemic-hypoglycemic clamp, activates the CB. This raises a couple of questions, as follows: (1) is the CB’s participation in the counterregulatory response to hypoglycemia dependent on insulin levels?; if not, (2) can we see the same physiological responses to low glucose independently of insulin?; (3) can CB glomus cells sense changes in insulin levels?; if so, (4) can CB malfunctions induce insulin resistance? These inquiries are particularly important in the context of the pathophysiology of type 2 diabetes, in which hyperinsulinemia, an imbalance in insulin/glucagon levels, and insulin resistance are associated with the development and progression of the disease [124,125,126].

A series of studies have been conducted to ensure the effects of CB on the counterregulatory response to hypoglycemia in an insulin-controlled condition. In dogs, high-intensity exercise promoted the progressive reduction of arterial glucose with no changes in insulin levels [127]. The bilateral resection of CBs caused a more pronounced and faster decline in glucose levels, and blunted the exercise-induced glucagon and norepinephrine responses, which corresponded to only 50% of the response in sham-surgery dogs. In humans, similar findings were observed in high-performance athletes submitted to two exercise sessions (65% peak oxygen consumption, up to 120 min) [128]. Blunting CB activation by the infusion of low doses of dopamine induced a greater fall in blood glucose levels, independently of circulating insulin [128]. In healthy individuals, the exposure to 3 h of IH (25 events/h, 5% O_2_, 3% CO_2_) increased circulating glucose levels with no differences in the oral glucose tolerance test, indicating no changes in the insulin sensitivity [129]. Taken together, these findings suggest that CB does play a role in the regulation of glucose metabolism independently of insulin. 

A second main point is whether CB glomus cells can sense insulin. Experiments with hypoglycemic hyperinsulinemic clamps suggest such a possibility. Insulin infusions increase minute ventilation and the rate of O_2_ consumption (VO_2_) in rats [111], and enhance HVR by about 108% in healthy individuals [107]. These findings were reproduced under euglycaemic conditions, using an intracarotid bolus of insulin without systemic hypoglycemia. Insulin increased minute ventilation in a dose-dependent manner in rats, and CB denervation abolished the insulin effects [130]. Dr. Sílvia Conde’s laboratory has shown that insulin receptors are expressed in CB cells, and they are phosphorylated when incubated with insulin [130]. Moreover, infusions of insulin activated a neurosecretory response in CB, increasing intracellular Ca^+2^ and enhancing the release of dopamine and ATP. Taken together, this evidence supports the premise that CB can be directly stimulated by insulin via insulin receptors expressed in CB glomus cells, independently of low glucose levels, mediating ventilatory and neurosecretory responses.

It is well-known that both insulin and CB are sympatho-modulators, and the prolonged exposure to hyperinsulinemia or tonic activations of CB may evoke SNS overactivation and metabolic dysfunction. CB activity and insulin demonstrate a bidirectional relationship. High levels of insulin may lead to exaggerated CB sympathetic responses by directly acting in glomus cells. On the other hand, long-term overactivation of CB may exacerbate SNS, leading to hyperinsulinemia and insulin resistance. Insulin increases sympathetic activity in both human and animal models under euglycemic conditions, measured by higher MSNA and the release of cathecolamines [131,132,133]. In 1962, Pereda and collaborators [134] showed that insulin infusion in the absence of hypoglycemia promoted an increase in arterial blood pressure and SNS activity in anesthetized dogs, and this sympatho-excitation was higher when insulin was administered into the carotid artery than systemically. This initial evidence suggested that peripheral insulin sensing may lead to an increased SNS response. In rats, high-fat diets (HFD) significantly decreased insulin sensitivity, with no changes in fasting blood glucose [130,135]. HFD-induced insulin resistance also caused the activation of CB, stimulating ventilatory responses and neurotransmitter release associated with an increase in SNS activity. This sympathetic overactivation was evidenced by an elevated mean arterial blood pressure, a 30% higher heart rate variability, and the augmented release of adrenal medulla catecholamines [130,135]. In humans, similar findings have been observed. In healthy adults, hyperinsulinemia under a euglycemic condition increased MSNA, and a low dose of dopamine attenuated the hyperinsulinemia-induced sympatho-excitatory response [136]. However, acute hyperoxia did not reverse the increased sympathetic activity induced by insulin, suggesting that the CB may actually act on insulin-mediating sympatho-excitation during chronic exposures to insulin [136,137,138], as observed in HFD animals [130,135]. In this sense, this first set of evidence suggests that insulin does activate the CB, and mediates its chronic physiological responses through the activation of the SNS, and that the CB may be a peripheral sensor of insulin-mediating sympathetic responses [29].

Alternatively, CB overactivation can impact on insulin secretion and insulin resistance indirectly, by inducing tonic sympatho-excitation. HFD in rats induces insulin resistance independently of blood glucose levels [130,135]. HFD animals showed a 120% increase in plasma insulin levels, and CB denervation treated the insulin resistance and restored the normal insulin levels [130], suggesting the possible role of the CB in mediating insulin secretion. One important factor to be highlighted here is that glucose homeostasis involves a very complex mechanism of regulation, comprising not only the insulin pathway. Adequate glucose levels are mainly maintained by the opposing actions of insulin and glucagon [139]. While insulin has an anabolic activity, inducing the insulin-dependent uptake of glucose and glycogenesis [140], glucagon is a catabolic hormone, responsible for the hepatic and renal gluconeogenesis that raises blood glucose levels [141]. Indeed, the CB appears to regulate glucagon secretion, and the ablation of the CB decreased basal glucagon secretion in dogs [106,127]. Our group showed that the CB is involved in the mechanisms of chronic IH-induced glucose intolerance and insulin resistance [56]. In our study, chronic IH increased fasting blood glucose and hepatic glucose output, but did not change the whole body glucose flux during the hyperinsulinemic euglycemic clamp. However, CB denervation did abolish the overall IH-induced effects on glucose metabolism, improving hepatic insulin signaling and preventing the hepatic sympathetic innervation induced by IH. Thus, our results support the evidences that the CB plays an important role in the regulation of insulin metabolism and the development of insulin resistance, with CB denervation having a beneficial effect on insulin sensitivity by improving insulin signaling in the liver, possibly via the regulation of hepatic sympathetic innervation.

Emerging evidence suggests that overactivation of the CB is implicated in metabolic dysfunction in type 2 diabetes. Two main points should be discussed: first, how does CB respond to hyperglycemia in a state of insulin resistance? Then, secondly, could insulin-induced CB overactivation be a predictor of type 2 diabetes development? It is known, as mentioned above, that IH induces hyperglycemia, which is abolished by the denervation of the CB [56]. However, high levels of glucose do not trigger CB overactivation. In healthy volunteers, insulin-induced hypoglycemia caused a robust increase in basal ventilation and HVR, but the hyperglycemic clamp did not reproduce the same strong effects on ventilation [107]. In agreement, Conde and collaborators [9] demonstrated that hyperglycemia, induced by the infusion of 25 mM of glucose, did not change CSN activity. Taken together, these data have indicated that hyperglycemia does not activate the CB, and that maybe hyperinsulinemia/insulin resistance is the key trigger of CB-mediated metabolic diseases, via sympatho-excitation. 

The CB is overactivated in prediabetic patients, and the variables related to CB chemosensitivity significantly correlate with fasting plasma insulin levels and Homeostatic Model Assessment for Insulin Resistance (HOMA-IR) [142]. Silencing CB activity via hyperbaric oxygen therapy significantly improved fasting blood glucose and glucose tolerance in patients with type 2 diabetes [143]. A recent report has also suggested that type 2 diabetic rats experience a high frequency shift in CSN and SNS neural activities, and the resection of the CSN normalizes SNS activity [144]. Taken together, this evidence suggests the following: (a) the CB directly senses blood insulin and acts in the balance of insulin/glucagon secretion; (b) insulin stimulates the CB and promotes physiological responses via sympatho-excitation; (c) the chronic overactivation of the CB increases tonic SNS activity, and elicits insulin resistance and dysmetabolism; and (d) insulin-mediated overactivation of the CB appears to be an early and functional predictor of type 2 diabetes.

### 4.3. CB Chemoreflex in Obesity: How to Dissociate the Metabolic Effects

Obesity is characterized by a complex and not fully-known pathophysiology, involving behavioral, genetic and physiological determinants [145]. Chemosensory alterations are frequently observed in obese patients, suggested by an increased ventilatory response to hypercapnia [146,147,148]. In rats, a reduced HVR has also been described in obese Zucker rats compared to lean controls, with body mass being a modest predictor of the ventilatory responses during hypoxia [149]. Differences in the ventilatory chemoresponsiveness have also been proposed as an explanation for the development of alveolar hypoventilation and obesity hypoventilation syndrome (OHS) in a subset of obese patients. However, the evidence concerning the ventilatory responses to hypoxia and hypercapnia in OHS patients is still contradictory, with some reports suggesting a reduced respiratory response [150] and others showing similar responses to controls [151]. In part, this lack of consistency could be attributed to the confounding effects of the underlying metabolic diseases. Excessive adiposity has been linked to impairments in glucose metabolism and insulin resistance, and arterial hypertension [152]. This combination of metabolic disturbances impairs the analysis of CB chemosensory activity in obese patients, since (as reviewed above) changes in glucose and insulin levels, and hypertension per se, are associated with the peripheral chemoreflex. As the relationship between CB function and the main components of metabolic syndrome were extensively discussed above, in the next section we will focus on the evidence of CB chemosensory activity in obesity-related hyperleptinemia and OSA.

#### Obesity, Leptin and OSA: A Trio for CB Activation

Obesity is associated with both the impaired signaling of the adipokine leptin [12] and the occurrence of sleep-disordered breathing [153]. Leptin is an adipocyte-produced hormone, responsible for suppressing appetite and enhancing the metabolic rate [154,155,156]. Leptin has also been shown to be a potent stimulator of breathing by centrally acting in the LepR^b^ expressed in the hypothalamus and medulla [157,158,159,160]. High plasmatic levels of leptin are observed in obesity; however, obese individuals are resistant to the metabolic and respiratory effects of leptin [12]. One of the causes of leptin resistance in obese subjects is attributed to a poor permeability of the blood–brain barrier to leptin, induced by impairments in the transport of leptin to the central nervous system [13,16,17,161,162]. Hyperleptinemia is also observed in patients with OSA [163,164,165,166,167], with some studies showing modest correlations between leptin and the apnea-hypopnea index [168,169]. OSA severity is also associated with body mass, in which the accumulation of fat adipose tissue, especially surrounding the upper airway, increases the risk of obstructions during sleep [153,170]. Taken together, these observations indicate that obesity, OSA and hyperleptinemia/leptin resistance are conditions that frequently co-exist in the same individual, suggesting that they could share a similar pathophysiology. 

Our laboratory has utilized mouse models of obesity, and shown that leptin is a powerful respiratory stimulant, which dramatically increases the hypercapnic response and HVR. Leptin-deficient *ob/ob* mice, hyperleptinemic and leptin resistant DIO mice, and New Zealand obese mice (NZO) hypoventilate during sleep, and retain CO_2_ [157,171,172,173], similarly to patients with OHS. This defect was reversed by leptin delivery beyond the blood–brain barrier by intracerebroventricular [160] or intranasal routes [174]. In addition, both leptin deficiency and leptin resistance have a detrimental effect on upper airway collapsibility, leading to OSA [171,172,173], which has also been relieved by leptin delivery to the CNS [160,174]. 

Leptin can also act peripherally, modulating CB chemosensory activity and stimulating breathing. Porzionato and collaborators [175] were the first group to identify the expression of leptin and leptin receptors in the CB. LepR^b^ was expressed in approximately 57% of type I cells in the human CB. Later on, Dr. John Ciriello’s laboratory demonstrated that leptin promotes the activation of glomus cells through LepR^b^, indicated by increased c-Fos gene expression and the phosphorylation of the signal transducer and the activator of transcription 3 (pSTAT3) [176,177]. We have shown that leptin increases the chemosensory inputs to the CB, and the systemic infusion of leptin increased CSN activity in response to hypoxia [97,98]. The subcutaneous infusion of leptin in lean mice, as well as the replacement of LepR^b^ in the CB of LepR^b^-deficient *db/db* mice, augmented minute ventilation during hypoxia and enhanced the HVR, while CB denervation abolished these leptin effects [98]. LepR^b^ expression in CB also increased ventilation during sleep [98]. The role of leptin in the regulation of HVR was confirmed, by other groups, in obese Zucker rats [178] and in Wistar rats under leptin infusion [179]. Interestingly, Ribeiro and collaborators [179] have shown that the respiratory effects of leptin were blunted with the introduction of HFD, suggesting a compromised ventilatory adaptation possibly induced by the development of leptin resistance. Taken together, this evidence indicates that leptin acts in the CB, activating LepR^b^ in the glomus cells to increase ventilation in response to hypoxia and during sleep, which could be a possible protective mechanism against sleep-disordered breathing. On the other hand, it is important to highlight that an overactivation of leptin signaling in the CB may have an opposite effect on sleep-disordered breathing, in which the excessive stimulation of CB by leptin may produce increases in ventilation, impairing breathing stability and inducing flow limitations during sleep. Our data suggest that leptin activates CB through the TRPM7 channels [96,97], which may play an important role in the regulation of leptin signaling in the chemosensory response and breathing stimulation. However, the molecular regulation of TRPM7 channels by leptin, as well as the involvement of the TRPM7 channels in the control of breathing, have not been investigated yet.

Obesity, OSA and hyperleptinemia are related to augmented SNS activity. As discussed above, chronic IH, as observed in OSA patients, increases the sympathetic tonus by inducing the functional plasticity of the CB chemoreflex [34,65]. On the other hand, leptin per se can also regulate sympathetic responses. Leptin regulates food intake and energy expenditure by stimulating SNS activity in the hypothalamus [180,181]. Sympathoactivation induced by leptin evokes thermogenesis in the brown adipose tissue, lipolysis, and increases in the metabolic rate [180]. In addition, LepR^b^ expression was identified in the caudal NTS, and intra-carotid injections of leptin increased the discharges of single units of NTS regions, suggesting that leptin could act as a mediator of the chemosensory circuit that potentiates the sympathoexcitatory responses [182]. As leptin can activate the CB, it has been proposed that the increased SNS activity induced by leptin is mediated by the activation of the peripheral chemoreflex [183]. In this sense, IH and leptin may share similar pathways to induce sympatho-excitation by acting in the CB. Thus, in obese patients, the combination of OSA and hyperleptinemia may have a synergistic effect on CB, causing the overactivation of CB afferent input and an exacerbated sympathetic response.

## 5. CB Manipulations: Therapeutic Targets for Metabolic Syndrome

### 5.1. CB Resection: Do the Benefits Outweigh the Costs?

Given the role of CB chemosensory activity in the development of cardiometabolic dysfunction, some investigators had proposed that the surgical resection of the CB could be a therapy for metabolic syndrome. As mentioned above, different protocols of CB resection or denervation by CSN dissection have been shown to have beneficial effects on metabolism. The ablation of CB activity reduces blood pressure [57,58,96,184] and normalizes the glucose and insulin levels [56,127,130] in animal models. In humans, the surgical resection of CB is a therapeutic approach for treating CB tumors [185]. In a retrospective study, Fudim and collaborators [92] showed that CB tumor resection in hypertensive patients reduced systolic blood pressure and pulse pressure independently of the use of anti-hypertensive medications. Nevertheless, these effects were only found in the first 30 days after the surgery, and the significant benefits of CB ablation were no longer observed in long-term analysis. In another study, unilateral CB resection decreased the ambulatory systolic blood pressure up to 6 months after the surgery, but it returned to basal values after a year [186]. This lack of any long-term effects of CB ablation on blood pressure was associated with acute-only reductions in MSNA. The short-term effects of CB resection have also been observed in studies of glucose metabolism. CB-resected patients (13 ± 5 years since unilateral CB resection) show a normal counterregulatory response to hypoglycemia, indicated by similar increases in plasma catecholamines, cortisol and glucagon, compared to non-resected controls [187]. Taken together, this evidence suggests that CB ablation does treat cardiometabolic diseases when examined shortly after the surgical manipulation. On the other hand, CB resection does not show long-term benefits in the treatment of hypertension and glucose dysfunction, highlighting possible adaptive mechanisms. Putative mechanisms include the neuroplasticity of the afferent chemosensory input, such as an upregulation of chemoreceptors in the aortic body, and the efferent response, including the facilitation of phrenic motor neurons [188]. 

The safety of CB resection is also an important point in ensuring its therapeutic efficacy. As reviewed by Paton and collaborators [59], CB resection, both unilateral and bilateral, does not appear to increase the risk of mortality in patients with HF and chronic obstructive pulmonary disease (COPD). This review also showed that the general frequency of adverse events was less than 3% in over 5000 patients. However, the safety of CB resection in patients with metabolic syndrome remains unclear. CB resection induces hypoventilation and increases the partial pressure of carbon dioxide (PaCO_2_) under normoxic conditions [189]. CB ablation reduces or almost eliminates HVR [190], which is a primary compensatory and protective mechanism against hypoxemia. A lack of increased ventilation during hypoxic challenges is not only observed acutely, but it can remain up to 4 years after CB ablation, as shown in a case report by Dahan and collaborators [191]. Thus, the permanent lack of ventilatory responses to hypoxic conditions could exacerbate the cardiometabolic consequences of sleep-disordered breathing. An aggravation of OSA severity was one adverse events reported by Narkiewicz and collaborators [186]. In one patient, the apnea-hypopnea index rose from 20 to 74 events/h, 3 months after the CB removal. Other investigators have also suggested the autonomic consequences of CB resection, indicated by an impaired blood pressure response to hypoglycemia, even with a normal baroreflex function [192]. Taken together, these findings suggest that CB resection impairs hypoxic ventilation, which can be detrimental in patients with sleep-disordered breathing, and may induce autonomic imbalance. Thus, the search for novel therapeutic approaches, focusing on pharmacological manipulations of the CB, is warranted.

### 5.2. Molecular Targets in the CB: Promising Pharmacology for Metabolic Syndrome

**Figure 2:**
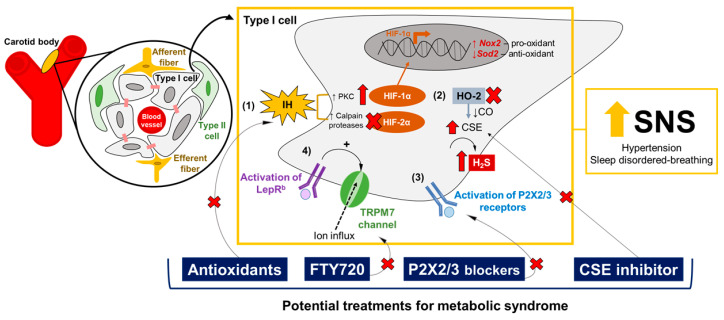
Proposed mechanisms involved in the excitation of the sympathetic nervous system (SNS) evoked by overactivation of the carotid body’s (CB) chemosensory response, and potential pharmacological targets in the CB for the treatment of metabolic syndrome. These pathways include (**1**) the transcriptional modifications of hypoxia-inducible factors 1 and 2 alpha (HIF-1α/2α) during hypoxia, (**2**) the balance of gasotransmitters in the CB, (**3**) the activation of purinergic receptors P2X2/3, and (**4**) the leptin-mediating transient receptor potential melastatin 7 (TRPM7) channels’ activation. IH: intermittent hypoxia; LepR^b^: long-isoform of leptin receptors; PKC: protein kinase C; CSE: cystathionine-γ-lyase; H_2_S: hydrogen sulfide; HO-2: hemeoxygenase-2; CO: carbon monoxide.

Several molecular pathways are implicated in the regulation of O_2_ sensing in the CB. The manipulation of these molecular pathways in the CB has shown overall improvements in cardiometabolic parameters, suggesting potential pharmacological targets for metabolic syndrome. Some of these molecular targets include: (1) HIF-1α and HIF-2α, (2) gasotransmitters CO and H_2_S, (3) purinergic receptors, and (4) leptin-mediated TRPM7 channels (Figure 2).

- HIF-1α and HIF-2α pathways: Dr. Gregg Semenza’s and Dr. Nanduri Prabhakar’s laboratories have provided plenty of evidence that ROS are involved in the overactivation of CB induced by IH [55,99,193,194]. IH augments the production of ROS in the CB, especially increasing the superoxide anion levels and the consequent elevation of hydrogen peroxide (H_2_O_2_) [195,196]. IH-induced ROS production in the CB occurs through different mechanisms, such as the activation of NADPH oxidase 2 [196] and the inhibition of superoxide dismutase 2 (Sod2) [197]. The transcriptional regulation of IH-induced oxidative stress in the CB is mainly governed by the balance between HIF-1α and HIF-2α signaling [99,193,194,198]. Both are heterodimeric transcription factors involved in oxygen homeostasis [198], and are expressed in the CB’s glomus cells [199]. However, HIF-1α and HIF-2α have opposite functions in the CB: HIF-1α activates Nox2 gene expression, the gene encoding to the pro-oxidant enzyme NADPH oxidase 2, while HIF-2α promotes the transcription of the Sod2 gene, inducing the expression of the Sod2 enzyme responsible for catalyzing the conversion of superoxide to hydrogen peroxide [193,194,197,198]. IH increases HIF-1α levels, and induces the degradation of HIF-2α via Ca^+2^-dependent protein kinase C (PKC) and calpains proteases, respectively [197,200]. Pharmacological and genetic manipulations of the HIF-1α and HIF-2α pathways in the CB have shown promising effects on the regulation of the CB’s chemoreflex, ventilatory stability and blood pressure [201,202] (Table 1), through a mechanism of mutual antagonism [203]. Therefore, drugs that selectively inhibit HIF-1α or upregulate HIF-2α in the CB, modulating the expression of NADPH oxidase 2 and Sod2 enzymes, could be potential targets for hypertension and OSA.
- Gasotransmitters: Dr. Nanduri Prabhakar’s laboratory have shown that CO and H_2_S may mediate the CB’s chemosensory response to hypoxia [194,204]. CO, which is generated by hemeoxygenase-2 (HO-2), inhibits CB activity [205], while H_2_S is catalyzed by the enzyme CSE and stimulates the hypoxic response in the CB [204]. The pharmacological blockade of CSE reduces H_2_S levels and normalizes breathing and blood pressure [53,204,206] (Table 1). Therefore, targeting the upregulation of HO-2 and the downregulation of CSE, with consequent increases of CO and the reduction of H_2_S generation in the CB, may be a pharmacological intervention for metabolic syndrome, attenuating hypertension and OSA. Moreover, Yuan and collaborators [207] have shown that IH-evoked ROS inactivates HO-2 in the CB, increasing the generation of H_2_S, suggesting that the balance of gasotransmitters could also be involved in the HIF-1α and HIF-2α signaling pathways in the CB [194].
- Purinergic receptors: The peripheral chemoreflex involves multiple excitatory postsynaptic responses, and ATP is the main neurotransmitter responsible for the activation of petrosal chemoreceptive terminals by binding to P2X2/3 receptors [208,209,210]. P2X2/3 receptors are also expressed in the glomus cells, promoting the excitation of the CB units induced by hypoxia and hypercapnia [211]. Dr. Julian Paton’s laboratory has shown that purinergic signaling may play a crucial role in the generation of aberrant chemoreflex responses in the CB, leading to hypertension and sleep-disordered breathing. Hence, the pharmacological antagonism of purinergic receptors in the CB has been proposed as a potential pharmacological approach to normalizing blood pressure and breathing stability [212,213] (Table 1).
- Leptin-mediated TRPM7 channels: Our group has shown that leptin acts in the CB and increases CSN activity to increase blood pressure through the activation of TRPM7 channels [96,97]. Considering that leptin exerts multiple functions, regulating the metabolic rate and energy expenditure [154,155,156], and that leptin resistance is often observed in obese patients [13,16,17], we propose that the pharmacological blockade of the TRPM7 channels in the CB could be a potential and more feasible therapy for metabolic syndrome. In this scenario, the administration of FTY720 to the CB, a potent inhibitor of the TRPM7 channels, has shown promising effects on the control of blood pressure [96] (Table 1). Our group has also demonstrated that leptin is a potent stimulator of ventilation and HVR, via its activating of the CB’s glomus cells as well as CSN activity [98]. Thus, we hypothesized that the blockade of TRPM7 channels with FTY720 in the CB could also be a treatment for sleep-disordered breathing (Table 1).

## 6. Conclusions

The CB’s function is not merely related to sensing blood gases and pH, but it also plays a crucial role as a peripheral sensor of metabolites and hormones, regulating metabolism and being intimately involved in the pathophysiology of cardiometabolic diseases. We extensively reviewed the evidence of the CB being implicated in the regulation of blood pressure and glucose metabolism, acting on the counterregulatory responses to hypoglycemia and mediating insulin/glucagon secretion. The CB induces sympatho-excitation, which links an abnormal chemosensory response to the pathogenesis of obesity, insulin resistance, type 2 diabetes, hypertension and OSA. The manipulation of molecular pathways involved in the O_2_ sensing function of the CB has shown overall benefits in the treatment of metabolic syndrome, normalizing blood pressure and the autonomic dysfunctions of hypertensive subjects, and reducing sleep-disordered breathing. Nevertheless, evidence for the efficacy of pharmacological interventions targeting the CB in diabetes, obesity and dyslipidemia is lacking. Therefore, further investigation of pharmacological interventions in the CB, targeting metabolic outcomes, is warranted, particularly in patients with augmented chemosensitivity.

## Figures and Tables

**Table 1:** Potential pharmacotherapies in the carotid body (CB) for the treatment of metabolic syndrome.

Molecular Target	Manipulation	Potential Drug	Main Outcomes	Key Evidence
HIF-1α and HIF-2α signaling	Downregulation of HIF-1α and NADPH 2 oxidase;Upregulation of HIF-2α and Sod2	Not identified	Hypertension and SDB	1- HIF-2α^+^/^−^ mice have an increased HIF-1α expression and consequent activation of Nox2 transcription in CB, while the reduced cellular oxidation in HIF-1α^+^/^−^ mice is caused by an elevated expression of HIF-2α and Sod2 gene [203]; 2- HIF-2α^+^/^−^ mice had decreased gene expression of Sod2, increased HVR, breathing instability and post-sigh apneas, and elevated blood pressure. The treatment with the antioxidant MnTMPyP abolished the autonomic and ventilatory dysfunctions [201]; 3- HIF-1α^+^/^−^ mice are resistant to IH-induced LTF in CB, augmented HVR, and hypertension [202].
Gasotransmitters	Blockade of CSE	2-Arylidene Hydrazinecarbodithioates		1- 2-Arylidene Hydrazinecarbodithioates is a potent and selective inhibitor of CSE [214]; 2- CSE^−^/^−^ mice have a blunted CB sensory activity and impaired HVR [204]; 3- _L_-PAG, a blocker of CSE, reduces H_2_S levels in the CB by 55% and abolishes hypoxia-evoked H_2_S generation [204]; 4- _L_-PAG reduced the release of catecholamines from adrenal medulla [204], decreased apneas in HO-2^−^/^−^ mice in a dose-dependent manner [206], and normalized blood pressure in SH rats [53].
Purinergic system	Antagonism of P2X2/3 receptors	AF-219 and AF-454		1- AF-219 and AF-454 are highly selective P2X3 receptor antagonists and AF-219 was clinically tested to treat patients with refractory chronic cough [215]; 2- In SH rats, AF-219 administered to the CB reduced the blood pressure in a dose-dependent manner and decreased the sympathetic tone [212]; 3- Systemic administration of AF-454 blunted the HVR and reduced the occurrence of apneas in newborn rats [213].
Leptin-TRPM7 axis	Blockade of TRPM7 channels	FTY720		1- FTY720 is a fingolimod that downregulates sphingosine-1 phosphate receptor and is an FDA approved drug for treating multiple sclerosis [216]; 2- FTY720 is a potent inhibitor of TRPM7 channels [217,218] and prevents the leptin-induced increase in TRPM7 currents in glomus cells [96]; 3- FTY720 administered to the CB abolished hypertension in C57BL/6J mice under leptin infusion [96]; 4- Leptin increases ventilation and HVR. FTY720 could stabilize breathing and treat SDB (?).

HIF-1α/2α: hypoxia-inducible factors 1 and 2 alpha; HVR: hypoxic ventilatory response; MnTMPyP: manganese (III) tetrakis (1-methyl-4-pyridyl) porphyrin pentachloride; IH: intermittent hypoxia; LTF: long-term facilitation; CSE: cystathionine-γ-lyase; SDB: sleep-disordered breathing; H_2_S: hydrogen sulfide; _L_-PAG: _L_-propargylglycine; HO-2: hemeoxygenase-2; SH: spontaneously hypertensive; TRPM7: transient receptor potential melastatin 7 channels; FDA: Food and Drug Administration.
